# Corneal Endothelial Cell Damage Following Bee Stings: A Report of Two Cases

**DOI:** 10.7759/cureus.92397

**Published:** 2025-09-15

**Authors:** Yoshihiro Yoshitani, Hideki Fukuoka, Chie Sotozono

**Affiliations:** 1 Department of Ophthalmology, Kyoto Prefectural University of Medicine, Kyoto, JPN

**Keywords:** bee venom, bullous keratopathy, corneal bee stings, corneal edema, corneal endothelial cell damage, corneal infection, honeybee, lasik-related infection, ocular trauma, paper wasp

## Abstract

Corneal bee stings cause inflammation through direct venom toxicity and type I hypersensitivity reactions that can lead to endothelial cell damage. In severe cases, these injuries can progress to bullous keratopathy, a condition that necessitates corneal transplantation. Here, we reported two cases in which transparent healing was achieved through steroid treatment, yet subsequent corneal endothelial cell damage ensued.

In the initial case, an 81-year-old male sustained a sting in the left eye from a honeybee. Initial visual acuity was 20/17 in the right eye and 20/33 in the left eye. A preliminary evaluation of the patient’s eye revealed the presence of corneal epithelial damage, Descemet’s membrane folds, and corneal edema measuring approximately 2 mm in the inferonasal peripheral cornea. Following treatment with topical steroids and oral betamethasone from day one, the cornea exhibited a transparent healing response with visual acuity improving to 20/33 in the affected eye. However, a significant decrease in endothelial cell density was observed, measuring at 1,306 cells/mm² in the affected eye, compared to the normal density of 2,677 cells/mm² in the contralateral eye. In the second case, a 49-year-old male with a history of laser-assisted in situ keratomileusis surgery was stung in the right eye by a paper wasp (*Polistes*). Initial visual acuity was 20/40 in the right eye and 20/13 in the left eye. A preliminary evaluation revealed the presence of a corneal epithelial defect measuring approximately 3 mm in diameter in the superior cornea, Descemet’s membrane folds, corneal edema extending across the entire cornea, and posterior corneal deposits. Following treatment with topical and systemic steroids, a state of transparent healing was achieved with final visual acuity of 20/20 in the affected eye; however, endothelial cell density was significantly reduced to 1,243 cells/mm² in the affected eye compared to 2,213 cells/mm² in the unaffected eye. Contact specular microscopy revealed a particularly severe reduction at the sting site (1,151 cells/mm²) compared to the peripheral area (3,212 cells/mm²).

The administration of steroids, both locally and systemically, has been demonstrated to be an effective treatment for corneal bee stings. Notwithstanding the efficacy of transparent healing, there is the possibility of a decrease in corneal endothelial cell density. The findings of this study indicate the necessity of meticulous follow-up procedures, even in cases that are initially treated with a conservative approach.

## Introduction

Corneal bee stings may result in both toxic and allergic inflammatory responses due to the action of bee venom. These responses can lead to severe outcomes, including bullous keratopathy, which necessitates corneal transplantation, or milder effects such as epithelial injury. The severity of these reactions can be classified as mild to moderately intense [1-3].

Bee venom is composed of various components, including amines such as histamine and serotonin, low-molecular-weight peptides, and hydrolytic enzymes, including protoenzymes that are capable of destroying tissues [4]. Honeybees (*Apis mellifera*) are considered to be the least harmful species, followed by Polistes paper wasps (*Polistes sp.*) and Vespa hornets (*Vespa sp.*). The venom of honeybees is considered the least dangerous, with its potency increasing with each species. Paper wasps have the capacity to inflict multiple stings, while hornets possess a substantial quantity of venom. However, both wasps and hornets exhibit a pattern of increasingly severe consequences following repeated stings. Consequently, the toxicity of venom escalates from honeybees to paper wasps and subsequently to hornets [5].

Local and systemic steroid therapy is the prevailing approach toward rehabilitation from corneal bee stings inflicted by honeybees and paper wasps [6,7]. The severity of hornet stings frequently necessitates the implementation of more extreme techniques, such as immediate anterior chamber irrigation or vitrectomy. In contrast, less aggressive measures are occasionally necessary for honeybees and paper wasps [5].

Recent studies have provided additional insight into the pathophysiology of corneal bee stings. Transcriptomic analysis has demonstrated that bee venom induces significant changes in corneal gene expression related to inflammation and tissue repair mechanisms [4]. Furthermore, comprehensive reviews of ocular manifestations and clinical outcomes in corneal bee sting injuries have highlighted the variability in presentation and prognosis [1].

The following case studies are presented to illustrate instances wherein the healing process of corneal lesions has been hindered by the administration of steroids, despite the implementation of treatment modalities aimed at facilitating such healing. These cases demonstrate the occurrence of damage to corneal endothelial cells in the absence of successful resolution of the underlying corneal lesions.

## Case presentation

This study presents two consecutive cases of corneal bee stings treated at our institution during the summer months between July 2023 and September 2023. Both cases were managed with steroid therapy and achieved transparent healing, yet demonstrated significant endothelial cell loss on follow-up.

Case 1

**Figure 1:**
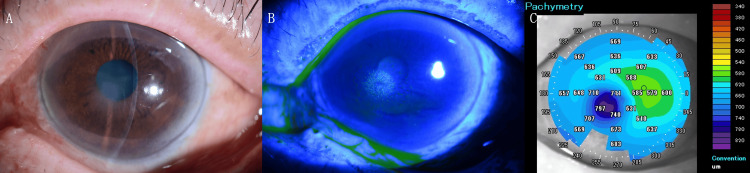
Clinical and imaging findings at initial presentation. (A) Slit-lamp photograph of the left eye showing localized Descemet’s membrane folds and corneal edema measuring approximately 2 mm in the inferonasal peripheral cornea at the site of the sting. (B) Fluorescein staining image demonstrating a localized corneal epithelial defect in the inferonasal region. (C) Anterior-segment optical coherence tomography confirming the presence of corneal edema in the inferonasal peripheral area.

An 81-year-old male patient sustained a sting on the left eye from a honeybee. The patient subsequently sought consultation with an ophthalmologist in his immediate vicinity. The patient noted a decline in his vision and corneal clouding and was referred to our hospital the following day. His best-corrected visual acuity was 20/17 in the right eye with a -1.00 D cylinder at axis 90 and 20/33 in the left eye with a +0.25 D sphere and -1.25 D cylinder at axis 90. A thorough examination of the left eye revealed the presence of a corneal epithelial defect, Descemet’s membrane folds, and corneal edema measuring approximately 2 mm in the inferonasal peripheral cornea (Figure 1).

The previous physician had prescribed the following medication regimen: four applications of gatifloxacin eye drops and four applications of 0.1% betamethasone eye drops, followed by the application of betamethasone eye ointment once daily. The patient was also instructed to apply betamethasone and d-chlorpheniramine maleate in the doses equivalent to 0.25 mg of betamethasone once daily.

**Figure 2:**
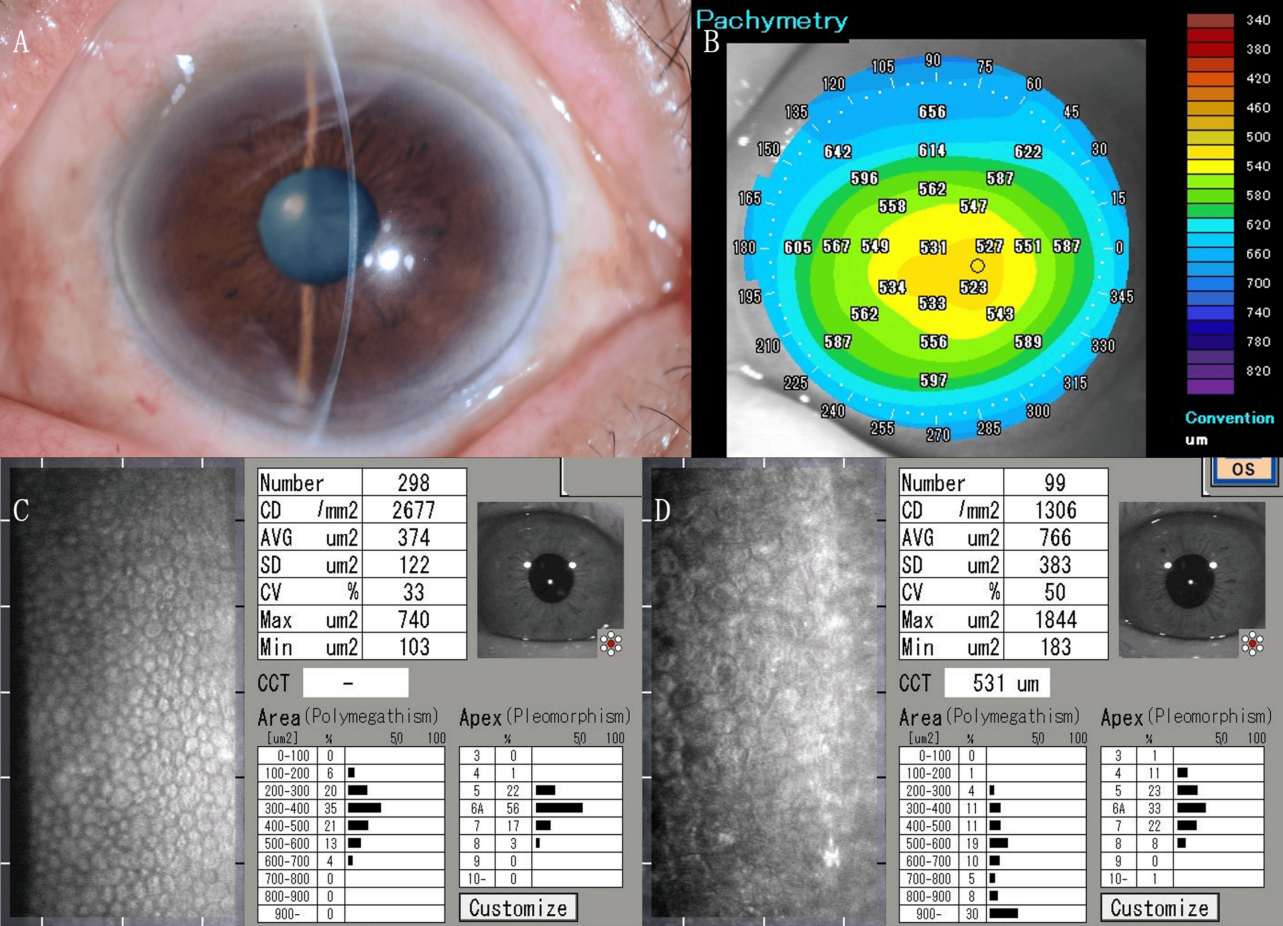
Clinical and imaging findings after treatment. (A) Slit-lamp photograph of the left eye demonstrating transparent corneal healing at day three post-treatment.(B) Anterior-segment optical coherence tomography in pachymetry mode showing resolution of corneal edema at day three. (C) Specular microscopy of the right eye at day three. (D) Specular microscopy of the left eye at day three.

Three days after our treatment, the patient’s left visual acuity exhibited 20/33 (with a +1.00 D spherical lens and a -1.25 D cylindrical lens at axis 90°). The change in spherical power while maintaining the same visual acuity line likely reflects corneal edema-induced refractive changes during the healing process. The recovery process was observed to include corneal epithelial sloughing, Descemet’s membrane folds, and corneal edema. A complete recovery was observed in the cornea, which exhibited transparent healing. However, a decrease in corneal endothelial cell density was noted. Specular microscopy revealed a reduction in cell density to 1,306 cells/mm² in the left eye, compared to 2,677 cells/mm² in the right eye (Figure 2).

The patient was evaluated on day three post-injury, at which time the above findings were documented. Specular microscopy was performed at this visit, revealing a significant endothelial cell density reduction. Despite scheduled follow-up appointments, the patient was subsequently lost to follow-up and did not return for further evaluation. Therefore, long-term outcomes beyond day three are unavailable for this case.

Case 2

A 49-year-old male sustained a sting from a paper wasp in three locations, including the right eyelid, head, and arm, while engaging in gardening activities. He was administered a methylprednisolone 125 mg infusion and an adrenaline 0.5 mg intramuscular injection for anaphylaxis at a local clinic, after which he was transferred to our hospital. The patient had undergone laser-assisted in situ keratomileusis (LASIK) surgery at the age of 28.

**Figure 3:**
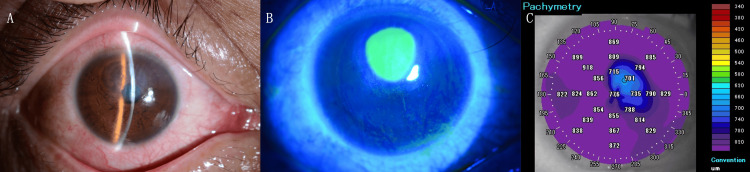
Clinical and imaging findings at initial presentation. (A) Slit-lamp photograph of the right eye revealing diffuse corneal edema, Descemet’s membrane folds, and posterior corneal deposits. (B) Fluorescein staining image showing a localized corneal epithelial defect measuring approximately 3 mm in diameter in the superior cornea. (C) Anterior-segment optical coherence tomography demonstrating marked corneal edema across the entire corneal thickness.

Upon thorough examination, the patient demonstrated a visual acuity of 20/40 in the right eye and 20/13 in the left eye, both without any corrective lenses. A thorough ophthalmological examination revealed a corneal epithelial defect measuring approximately 3 mm in diameter in the superior cornea of the right eye, accompanied by Descemet’s membrane folds, corneal edema affecting the entire thickness of the cornea, and corneal endothelial deposits (Figure 3). The treatment regimen was initiated with the administration of topical levofloxacin 0.5% eye drops six times daily, 0.1% betamethasone eye drops at the same frequency, and ofloxacin eye ointment four times daily.

**Figure 4:**
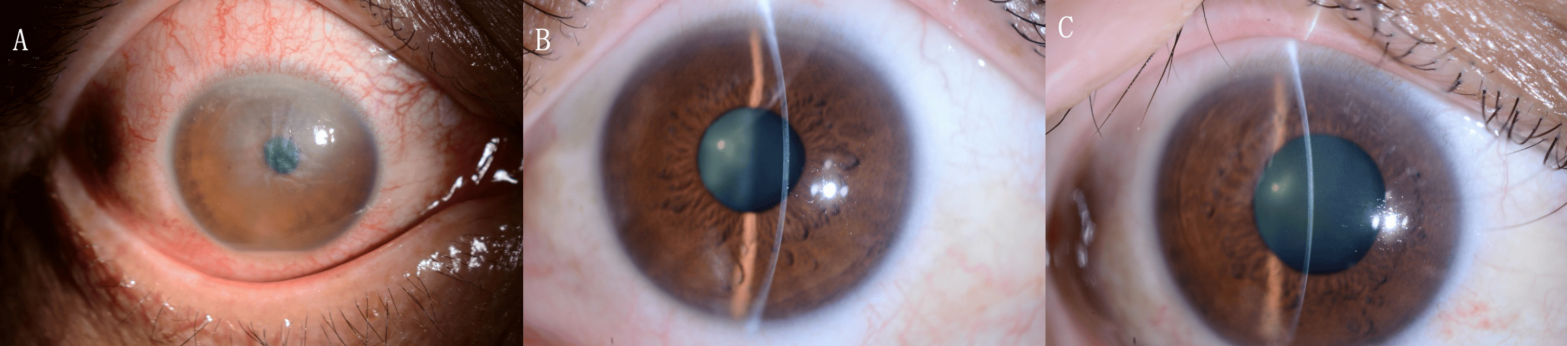
Clinical course of the right eye on slit-lamp examination. (A) Two days after treatment initiation: presence of hypopyon in the anterior chamber and edema of the laser-assisted in situ keratomileusis flap. (B) Nine days after treatment: noticeable improvement in corneal edema. (C) One month after treatment initiation: transparent corneal healing achieved.

Two days after the commencement of treatment, the patient exhibited a unilateral decline in visual acuity to 20/67. A thorough clinical evaluation revealed the presence of corneal edema, LASIK flap edema, and hypopyon in the anterior chamber. The patient was administered atropine eye drops and a 2 mg betamethasone infusion for a period of two days, in conjunction with a course of 10 mg of oral prednisolone. Following a nine-day treatment period, the patient exhibited signs of improvement in corneal edema, which led to the discontinuation of oral prednisolone. As indicated by the patient’s improved 20/20 visual acuity in the right eye (with -0.50 D spherical, -0.75 D cylindrical lens at axis 10°), measured one month after treatment initiation, the treatment was successful (Figure 4).

**Figure 5:**
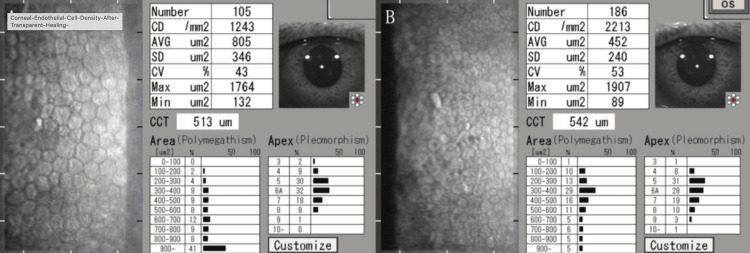
Corneal endothelial cell density at three months after transparent healing. (A) Non-contact specular microscopy (Tomey EM-3000, Tomey Corporation, Nagoya, Japan) of the right eye (injured eye) at three months post-treatment showing reduced endothelial cell density compared to the unaffected left eye. (B) Non-contact specular microscopy of the left eye at three months post-treatment for comparison.

Transparent healing was achieved; however, non-contact specular microscopy (Tomey EM-3000, Tomey Corporation, Nagoya, Japan) showed a reduction in corneal endothelial cell density to 1,243 cells/mm² in the right eye compared to 2,213 cells/mm² in the left eye (Figure 5). Contact specular microscopy revealed a particularly severe reduction at the sting site (1,151 cells/mm²) compared to the peripheral area (3,212 cells/mm²).

Extended follow-up at nine months demonstrated maintained endothelial cell density of 1,243 cells/mm² with excellent visual recovery to 20/13 with cylinder correction of -0.75 D at axis 10°. Intraocular pressure remained within normal limits at 9.7 mmHg. No late complications, including cataract progression or secondary glaucoma, were observed during the extended follow-up period.

## Discussion

Typically, corneal bee stings result in the stung cornea having accompanying subconjunctival or ciliary edema, along with some degree of conjunctival or ciliary hyperemia. However, they can potentially result in more severe complications, including iris atrophy, hemorrhage of the anterior chamber, traumatic cataract, subluxated lens, bullous keratitis, and even endophthalmitis [8-10]. The literature documents a comprehensive spectrum of complications that can occur, from mild anterior segment inflammation to vision-threatening conditions [3].

The literature suggests that various species of stinging insects produce different clinical manifestations and require different management approaches. Previous studies have specifically compared outcomes between hornet and paper wasp stings, finding significant differences in severity and prognosis [5]. Paper wasps and hornets have been observed to be effective in cases of severe wounding. Advanced forms of treatment are frequently necessary for these patients, including immediate surgery and, in certain cases, vitrectomy. In contrast to the treatment of hornets, local and systemic steroid therapy are the most aggressive approaches taken for honeybee and paper wasp stings [1,5].

The cases presented in this report involved successful management of honeybee and paper wasp stings with topical and systemic steroids, resulting in transparent corneal healing without the need for invasive interventions such as anterior chamber irrigation. This approach aligns with recommendations in the literature advocating for conservative management with steroids as a first-line treatment for isolated corneal bee stings [1].

However, despite achieving transparent corneal healing, both cases demonstrated significant decreases in corneal endothelial cell density. This finding is particularly noteworthy and is consistent with observations from previous research documenting toxic effects on corneal tissues following bee sting injuries [6]. Our observation of localized reduction in endothelial cell density at the site of the sting in Case 2 (1,151 cells/mm² at the sting site versus 3,212 cells/mm² in the peripheral area) suggests direct toxic damage to endothelial cells by the venom components.

The reduction in endothelial cell count, despite apparent clinical improvement, underscores the importance of comprehensive evaluation and long-term follow-up in bee sting injuries. Previous reports have documented cases of corneal bee stings that showed initial improvement without treatment [8]; however, our findings suggest that subclinical endothelial damage may occur even in cases with favorable clinical outcomes, necessitating careful monitoring for potential late complications.

In the context of our second case, the pre-existing LASIK surgery complicated the clinical picture, with the development of LASIK flap edema during the inflammatory phase. This highlights the potential for bee sting injuries to exacerbate conditions in corneas with previous surgical interventions, a consideration that has not been extensively documented in the literature.

Our findings of significant endothelial cell density reduction (51% and 44%, respectively) align with previous reports documenting persistent endothelial damage despite successful steroid treatment. Gürlü and Erda reported a substantial decrease in corneal endothelial cell density one year after bee sting injury despite comprehensive steroid therapy [11], while Gudiseva et al. demonstrated that 54.5% of patients achieved good visual outcomes (best-corrected visual acuity of >20/40) with medical therapy alone, consistent with our cases [1].

Unlike previous studies that included surgical interventions, our conservative steroid approach achieved comparable visual outcomes. The nine-month stability of endothelial cell density in Case 2 suggests that steroid therapy may stabilize the endothelium long-term. Our observation of localized endothelial damage at the sting site (1,151 cells/mm²) versus peripheral preservation (3,212 cells/mm²) provides anatomical evidence supporting direct venom toxicity as the primary mechanism.

These findings suggest that while bee venom causes immediate and irreversible endothelial damage, conservative steroid management can achieve excellent functional outcomes with stable long-term endothelial function. Gudiseva et al. emphasized that oral corticosteroid supplementation prevents serious vision-threatening complications [1], supporting our therapeutic approach. The heterogeneous pattern of endothelial loss supports targeted rather than diffuse corneal involvement, explaining why visual recovery is possible despite significant cell density reduction.

## Conclusions

The administration of steroids, both locally and systemically, has been demonstrated to be an effective treatment for corneal bee stings. Despite the efficacy of transparent healing through conservative treatment, there is the potential for a decrease in corneal endothelial cell density. This finding suggests that the toxic components of bee venom may cause lasting damage to corneal endothelial cells, even when clinical resolution appears complete. While conservative steroid therapy achieved excellent visual outcomes in our cases, the observed endothelial cell loss suggests that alternative treatment approaches warrant investigation. Future prospective studies comparing conservative management with early surgical intervention may help establish optimal protocols that preserve both visual function and endothelial cell integrity. It is imperative to exercise meticulous caution and pursue comprehensive follow-up measures, including specular microscopy, even in instances where the condition is addressed with a conservative approach and demonstrates apparent resolution.
